# The Infirmary Branch and Its Attractions for the Newly Qualified

**Published:** 1920-09-04

**Authors:** 


### THE POOR-LAW MEDICAL SERVICE.

### The Infirmary Branch and its Attractions for the Newly Qualified.

The Poor-law infirmary has always offered peculiar opportunities to the newly-qualified man for increasing his knowledge of the manifestations of disease and of methods of treatment. In it he finds patients suffering from diseases the course of which he has watched as a student in the wards of his hospital. But what is of greater importance he finds there also patients who are rarely admitted to the beds of a general voluntary hospital, but who will form a large part of those for whose care he will be responsible as a general practitioner.

### A Fertile Field for Research.

In every Poor-law infirmary one-third to one-half of the beds contain patients in the advanced stages of rheumatoid arthritis, gout, chronic bronchitis, heart disease, ulcers of the leg, and chronic nervous diseases; the terminal stages of inoperable malignant disease, and of phthisis; and the various pathological conditions that accompany old age. These are the patients whom he has been accustomed contemptuously to label chronic, and to regard as uninteresting and as hopeless from the point of view of treatment, because he has only seen them casually in the out-patient department, and has not come to know them as he knows the patients with acute and operable complaints. A few months' study of them in the wards of a Poor-law infirmary makes him see them from a different point of view. He begins to realise that his opinion of the hopelessness of alleviating or curing their condition springs from his own ignorance. He finds they present problems in diagnosis and in treatment as fascinating as any of the acute illnesses, and that there is here a field for research as yet almost unworked. As he gets beyond the stage when he lumps them all together as chronics he finds that each has an intensely interesting individuality, and that his sympathies are more and more bound up in them and in their progress. As he gets to know them, their patience under suffering, their oddities of speech, the pathos of their outlook upon life and health, their petulances and foibles appeal to him more than the sharp insistent cry of acute illness, and make calls upon his tact, ingenuity, and knowledge which he finds a special interest in satisfying. Many of these patients come to the infirmary worn out, dejected and hopeless, resigned to a bed-ridden existence. Once he has brought back the hope and the desire of improvement to such a patient; once he has succeeded in straightening the limbs and making movable the joints of one crippled with rheumatoid arthritis; once he has patiently re-educated a tabetic in the control of his inco-ordinate muscles; once he has cured a chronic ulcer which has resisted all treatment for years; he will have forged a bond of interest in chronic disease from which he will never escape. Such a success will give him as great a thrill of pleasure as helping a patient with pneumonia past the crisis, or curing a duodenal ulcer by a gastro-jejunostomy.

### The Type of Experience Gained.

The peculiar interest of medical work lies in the opportunities it affords of studying the reaction of humanity to adverse and benign influences. For the study of man in the prison-house of disease there is nothing to compare with the Poor-law infirmary. The short-sentence man—the man with acute disease—can be studied there or in the voluntary hospital. He passes through his term in a few days or weeks and his mental changes are comparatively slight and uninteresting. But the man to who long years of servitude have been dealt can be studied only in the Poor-law infirmary. He is the man who will degenerate into the peevish, hopeless invalid by the unremitting skill and care of those who nurse and treat him. Here is a Gilead that still cries, "Is there no physician? Why then is not the health of my people recovered?"

### Work in the Special Wards.

The newly-qualified man does not and cannot realise the wealth of interest and of possibilities this unregarded field of medicine holds. But the care and treatment of the chronic patient form only a small part of the work of most Poor-law infirmaries. And it is not only in this field that the Poor-law infirmary differs from and supplements the work in a voluntary hospital. There are always in them a large number of children suffering from diseases the newly-qualified man has had few opportunities of observing throughout their course. Children are admitted suffering from such infectious diseases as measles, German measles, chicken-pox, whooping-cough, and epidemic diarrhœa. The marasmic disorders of infancy can be studied here as they can be studied nowhere else, except in some of the special hospitals for children. All the large infirmaries have special wards for erysipelas, for scabies, for venereal disease, and for insanity. There are also usually a maternity ward, a pre-maternity ward, and a nursery for healthy children under three years of age and for suckling infants and their mothers. The amount of surgical work varies. In some very little operative surgery is done, but in most a large number of major operations—from two to five hundred—is done every year. The work in the wards for the insane is of special value to the newly-qualified man, because here he sees cases of incipient and doubtful insanity, and learns practically the details the law requires to be observed in the certification of the insane. To sum up, except for such infectious diseases as scarlet fever and diphtheria, the Poor-law infirmary has within its walls examples of every form of acute and chronic disease from infancy to old age. The wealth and interest of its clinical material are known only to those who have worked in one.

### A Hopeful Outlook.

Hitherto it has been necessary to warn those who thought of entering the indoor Poor-law medical

**The Poor-Law Medical Service—(*continued*).**

service that it was understaffed and underpaid, that there was no specialist staff, and that the equipment for diagnosis and treatment was inadequate. To-day in London and the large provincial towns the position is different. Stimulated by the insistent demands of their medical officers, and by the fear that the infirmaries will be transferred to other and more progressive authorities, and faced with the impossibility of obtaining medical officers under the old conditions, many Boards of Guardians have greatly improved the equipment of their infirmaries and the pay of their medical officers, and have begun to appoint a visiting staff of specialists. The infirmaries are still far behind the voluntary hospitals in the facilities at their command for diagnosis and treatment, but the outlook was never so hopeful as it is now. In London it is almost certain that the infirmaries will be transferred in a year or two to the London County Council. Then great changes may be expected. At present the infirmaries vary very greatly, and none of them can deal adequately with all the different classes of disease they contain. Under one authority some of them will be specialised, and all of them will be properly equipped and adequately staffed with resident and visiting specialists. London will then possess a group of municipal hospitals without a rival in the world. The medical service of these hospitals will be one which the newly-qualified man can be advised to join as a career, with the knowledge that good work will bring promotion to an administrative or specialist post. That is not the case to-day, because the only post in which a man can marry is that of the medical superintendent, and the chances of promotion to it are few, and too much a matter of luck.

### Present Emoluments and Conditions.

Leaving the possibilities of the future and dealing with the service as it is, the salary usually given to the junior medical officer is £300 a year with the usual residential allowances. The salary rises with promotion to about £500, which is the usual salary for an assistant medical superintendent. Salaries below these should not be accepted. The salary, including extra fees, for the medical superintendent varies from £700 to £1,200, usually with a house, laundry, heating, and light. No man should join the service, as at present organised, as a career, but to hold one of these posts for a few years before going into general practice is of great educational value. Each medical officer is responsible for the diagnosis and treatment of the patients in the wards allotted to him. He is thrown to a great extent upon his own resources and compelled to develop his confidence in himself and his powers of initiative. At the same time there are always accessible more experienced colleagues to whom he can turn for advice and assistance. Most special methods of investigation he will have to carry out himself. He will have many opportunities of using such aids to diagnosis as the ophthalmoscope, the laryngoscope, the sphygmo-manometer, $x$-rays and electricity, and the chemical and bacteriological examination of urine, sputum, gastric contents, etc. He will be required to give many anæsthetics, to do minor surgical operations, and to assist at major operations. The work is just that which will best fit him for the duties of a general practitioner. To the newly-qualified man looking out for a post in which he can maintain himself whilst enlarging his professional knowledge and experience the indoor poor-law medical service offers advantages not to be found in any other position.

### Beware of the Non-Separated Infirmary.

There is one warning to give. Poor-law infirmaries vary very much, even in our large towns, in equipment and in the character of the work done. The best of them are the separate infirmaries administered by a medical superintendent. With a very few exceptions infirmaries under the administration of the workhouse master should be avoided. The intending applicant should make inquiries as to the number of beds, the equipment, the number of admissions annually, the number of operations performed, the existence of special departments, and the strength of the nursing staff. The best infirmaries possess a pathological and bacteriological laboratory, $x$-ray apparatus, an electrical and massage department, visiting specialists, and an operating theatre. At least two or three hundred operations should be done, the number of admissions should be five or six times the average number of occupied beds, the nursing staff should be at least one nurse to six patients, and the medical officers should not be required to do the dispensing or any clerical work except clinical note taking.

